# School-Based Health Center Intervention Improves Body Mass Index in Overweight and Obese Adolescents

**DOI:** 10.1155/2013/575016

**Published:** 2013-03-26

**Authors:** Alberta S. Kong, Andrew L. Sussman, Carolina Yahne, Betty J. Skipper, Mark R. Burge, Sally M. Davis

**Affiliations:** ^1^Department of Pediatrics, University of New Mexico, Albuquerque, NM 87131-0001, USA; ^2^Department of Family and Community Medicine, University of New Mexico, Albuquerque, NM 87131-0001, USA; ^3^Department of Psychology, University of New Mexico, Albuquerque, NM 87131-0001, USA; ^4^Department of Internal Medicine, University of New Mexico, Albuquerque, NM 87131-0001, USA

## Abstract

Adolescents Committed to Improvement of Nutrition and Physical Activity (ACTION) was undertaken to determine feasibility of a school-based health center (SBHC) weight management program. Two urban New Mexico SBHCs were randomized to deliver ACTION or standard care. ACTION consisted of eight visits using motivational interviewing to improve eating and physical activity behavior. An educational nutrition and physical activity DVD for students and a clinician toolkit were created for use as menu of options. Standard care consisted of one visit with the SBHC provider who prescribed recommendations for healthy weight. Sixty nondiabetic overweight/obese adolescents were enrolled. Measures included BMI percentile, waist circumference, insulin resistance by homeostasis model assessment (HOMA-IR), blood pressure, triglycerides, and HDL-C levels. Pre- to postchanges for participants were compared between groups. Fifty-one students (mean age 15 years, 62% female, 75% Hispanic) completed pre- and postmeasures. ACTION students (*n* = 28) had improvements in BMI percentile (*P* = 0.04) and waist circumference (*P* = 0.04) as compared with students receiving standard care (*n* = 23). No differences were found between the two groups in blood pressure, HOMA-IR, triglycerides, and HDL-C. The ACTION SBHC weight management program was feasible and demonstrated improved outcomes in BMI percentile and waist circumference.

## 1. Introduction 

The prevalence of childhood obesity in the USA has tripled since 1980 and now affects 12.5 million school-age children and adolescents [1, 2]. Associated with this epidemic is the rising prevalence of metabolic syndrome among adolescents, particularly in obese teens (12.4 to 44.2%) [3]. The components of metabolic syndrome are typically described as a clustering of cardiometabolic risk factors that includes central adiposity, elevated blood pressure, dyslipidemia, and impaired glucose metabolism [4–6]. These derangements increase the risk for cardiovascular disease and type 2 diabetes [7], and weight loss through behavioral modification is the recommended first step in the prevention and treatment of metabolic syndrome [8].

 A challenge in delivering behavioral modification interventions is that adolescents seek medical care infrequently [9]. School-based health centers (SBHCs) that provide health care services to students on school campuses offer an opportunity to reach adolescents at a location where they spend a significant portion of their day [10]. SBHCs are designed to focus on the uninsured and underserved, and providers work with a large segment of the adolescent population during a key stage of development characterized by increased individuation and autonomy. The role of SBHCs in the battle against obesity has not been well investigated. We explored the feasibility of Adolescents Committed to Improvement of Nutrition and Physical Activity (ACTION), a SBHC weight management intervention for overweight, and obese students that was created and tested with two urban high schools. We hypothesized that overweight and obese students receiving ACTION would have a greater reduction in BMI percentile when compared with students receiving standard care. 

## 2. Methods

### 2.1. Study Design

 Two SBHCs were randomized to deliver either the intervention or standard care over the academic year of 2009-2010. This design was chosen to decrease contamination between the two groups. The SBHC clinician delivering the intervention was a family medicine nurse practitioner, and standard care was delivered by a family medicine physician. 

### 2.2. Participants

 Participants were recruited through classroom presentations made by the research team. Students were given study packets with health history survey and bilingual consent/assent forms which they reviewed and returned to the SBHCs. Pre- and posttest assessments were conducted at the SBHCs for all participants. The protocol was approved by the University of New Mexico (UNM) Human Research Protections Office and the Research, Development, and Accountability Department of both high schools. 

 Students were eligible to participate if they were in the 9th to 11th grades and had a BMI ≥85th percentile [11]. Exclusion criteria included BMI ≥40 kg/m^2^, previous diagnosis of diabetes, blood pressure in the range of stage 2 hypertension [12], antipsychotic or corticosteroid medications, or if the adolescent was not ambulatory. Withdrawal conditions included anorexia nervosa, bulimia nervosa, psychosis, suicidal ideation, hospitalization, and pregnancy.

**Figure 1:**
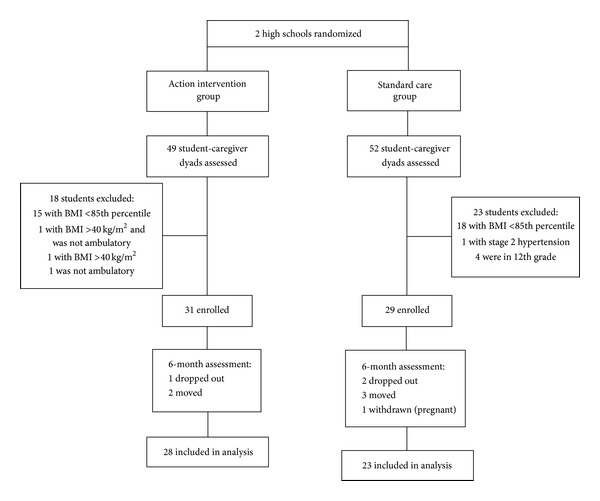
Diagram of randomization, enrollment, and attrition.

 A total of 60 students and their caregivers were enrolled (Figure 1). 28 of 31 student-caregiver dyads at the intervention high school and 23 of 29 student-caregiver dyads at the control high school completed pre- and postmeasures.

### 2.3. Study Groups

#### 2.3.1. Intervention Group

 ACTION, based on the Transtheoretical Model [13], included three primary components: (1) clinical encounters with the SBHC clinician every two to three weeks for a total of eight visits over one academic year, (2) use of motivational interviewing (MI) [14, 15], and (3) obesity risk reduction strategies from a toolkit that was cocreated with a community advisory group made of overweight and obese adolescents and their parents. The toolkit included a DVD and print materials to provide a “menu of options” during clinical encounters (Table 1). 

 The intervention SBHC provider received a two-day training workshop in MI. To determine competency, three pilot MI sessions were audiotaped and reviewed by the trainers prior to starting the intervention. Audio recording of the clinical visits followed by coaching occurred four times throughout the intervention period to ensure fidelity. 

 At the first visit, participants randomized to ACTION received the DVD, a DVD player and a summary of medical results (BMI, blood pressure, fasting glucose, and lipids) along with American Academy of Pediatrics (AAP) obesity prevention/treatment recommendations [11]. The first visit was dedicated to reviewing pertinent personal and family history, physical exam and laboratory findings, and an assessment of dietary and physical activity behavior. Feedback was provided to the adolescent about their status relative to national recommendations, and the adolescent's readiness to change was elicited. Participants were asked to review the DVD and to follow-up in two to three weeks with topics they would like to discuss. Subsequent visits were individually tailored to the adolescent's stage of change with the intention of moving towards goal setting for healthier eating and physical activity. 

 Students brought home a newsletter to their caregivers that included obesity risk reduction strategies for the home. After each visit, telephone updates were given to the caregiver, during which the SBHC clinician used MI to encourage caregivers to adopt the risk reduction strategies. 

#### 2.3.2. Standard Care Group (SCG)

The clinician was trained on the study protocol and procedural materials prior to initiating the trial. Participants in the SCG received one clinic visit at the beginning of the trial that was similar in content to the first visit of the intervention group except they were not given the DVD or DVD player. The AAP “Balance for a Healthy Life” booklet and medical results summary with AAP recommendations [11] were also provided to participants. 

### 2.4. Data Collection and Measurements

Anthropometric, blood pressure, biochemical, and behavioral measures were obtained at baseline (September-October 2009) and after the completion of the intervention (April-May 2010) in both groups.

#### 2.4.1. Anthropometric Measures

 Anthropometric measures were conducted by a registered dietitian. Height and weight were measured twice without shoes and averaged for analysis. Weight was measured to the nearest 0.1 kg on a strain-gauge digital scale (Secca Model 770) and height was measured to the nearest millimeter using a Schorr vertical measuring board. BMI was calculated as kg/m^2^. A CDC software program was used to calculate precise BMI percentiles based on participants' height (cm), weight (kg), sex, and age (months) [16]. Waist circumference was measured twice to the nearest millimeter with a steel tape and averaged.

#### 2.4.2. Blood Pressure

 Three seated blood pressures (BPs) were measured in the right arm with a Welch Allen aneroid sphygmomanometer (Skaneateles Falls, NY, USA) by a research pediatric nurse after 5 minutes of sitting and 1 minute between each measurement. The second and third measurements were averaged. BP percentiles for gender, age, and height were determined according to established guidelines [12].

#### 2.4.3. Biochemical Measures

 Blood samples were drawn after a 10-hour overnight fast. Samples were allowed to clot at room temperature for 15 minutes and centrifuged for 10 minutes. The serum fraction was aliquoted and stored at −80°C. Glucose was determined using the ACE Glucose Reagent from Alfa Wassermann Diagnostic Technologies, LLC (West Caldwell, NJ, USA). Insulin was measured using the Immulite/Immulite 100 Insulin assay from Siemens Healthcare Diagnostics Products Ltd. (Llanberis, Gwynedd, UK). Insulin resistance was calculated using the homeostatic model assessment insulin resistance index [17, 18]. Triglyceride was measured using the ACE Triglycerides Reagent Kit from Alfa Wassermann Diagnostic Technologies, LLC, and HDL cholesterol was determined using the Vitros Slide Technology kit. 

#### 2.4.4. Behavioral Measures (Dietary, Physical Activity, and Television Viewing)

 Dietary intake was assessed using the Youth/Adolescent Questionnaire (YAQ), a food frequency questionnaire designed for children ages 9–18 years [19]. Physical activity was assessed using the 3-Day Physical Activity Recall (3-D PAR) instrument and the RT3 Triaxial Research Tracker accelerometer (Stayhealthy Inc., Monrovia, CA, USA). Detailed instructions for completing the 3 D PAR were given using a standardized script. The 3 D PAR has been validated in adolescents based on concurrent observation with motion sensors [20, 21]. Standard scoring procedures [22] were used to estimate the number of 30-minute blocks per day participants spent in moderate or vigorous physical activity. RT3 Triaxial accelerometers recorded the intensity, frequency, and duration of participants' physical activity [23]. Written and verbal instructions for proper use of the RT3 were given to participants using a standardized script. They were instructed to wear the accelerometer on the right hip, except when sleeping or involved in water activities. Participants were reminded daily by either text message or phone call to wear their accelerometers. After one week of wear, accelerometer data were downloaded to a computer using a docking station provided with the RT3. Triaxial activity was captured as counts per minute, which represents the frequency and amplitude of acceleration events occurring over each minute of wear. Activity count data was converted to measures of physical activity intensity (metabolic equivalents or METs) using the RT3 proprietary equation. Moderate or vigorous physical activities were defined as ≥3 METs. Minimal wear requirement for a valid day was 10 hours, and four valid days of data per measurement wave were required to be included in the analysis. Television viewing time for each day of the week was recorded from an 11-item Television and Video Measure used in the Planet Health school-based intervention study [24]. Hours were appropriately weighted and summed to obtain a total hours-per-day viewing estimate.

#### 2.4.5. Process Measures

 Process evaluation was conducted to monitor how well the study was implemented. In addition to monitoring the fidelity of MI used by the intervention clinician, participant attendance, length of clinic visit, and participant satisfaction were collected in both groups. In the intervention group, phone contacts with caregivers were tracked.

### 2.5. Data Analysis

 Determination of sample size was based on previous reports of short-term weight loss interventions [25]. Twenty-one participants per group were estimated to have sufficient power (>0.80) with a significance level of 0.05 to detect a large effect size (Cohen's *d* = .84) between the two groups with respect to BMI changes [26]. We accounted for a 20% attrition rate and set to recruit 26 students per group.

 Baseline equivalence of conditions across demographics, BMI, and other outcome variables of interest were assessed with *t*-tests or Wilcoxon rank sum test on continuous items and *χ* ^2^ tests or Fisher's exact test on discrete items. Pre-post changes in BMI and other variables were compared between groups using two-sample *t*-tests for normally distributed data and Wilcoxon rank sum tests for skewed distributions using SAS (Cary, NC, USA). Analysis included participants who completed pre- and postassessments regardless of the number of clinical visits attended. 

## 3. Results

### 3.1. Participants

 There were no significant differences by group on demographic variables (Table 2) or baseline anthropometric measures (Table 3). The mean age of students in the intervention group was 15 ± 1 year compared to 14.6 ± 0.7 year for the SCG. The majority of the students were Hispanic and females. Reported family history of type 2 diabetes among first or second degree relatives was 52% and hyperlipidemia 43%. Enrolled caregivers were primarily mothers (70%) and were Hispanic with 41% having less than a high school graduate level education. Using the definition from the National Heart, Lung, and Blood Institute [27], six percent of enrolled students met criteria for metabolic syndrome defined as having three or more of the following: systolic ≥130 mmHg or diastolic ≥85 mmHg, fasting glucose ≥100 mg/dL, large waist circumference (male ≥102 cm, female ≥88 cm), low HDL cholesterol (male <40 mg/dL, female <50 mg/dL), or triglycerides ≥150 mg/dL. Forty-three percent had one component and ten percent had two components of the metabolic syndrome. Most of the students were in the action or maintenance stage of change.

### 3.2. Process Evaluation

 93% of students completed all eight clinical visits in the intervention group. Sessions averaged 47 minutes for the first session and 24 minutes for subsequent sessions. Student satisfaction scores averaged 4.4 for the intervention (0 = not satisfied, 5 = very satisfied). 

 88% of caregivers completed the feedback survey with an overall satisfaction mean score of 4.4 (0 = not satisfied, 5 = very satisfied). Caregivers of twenty-four children (86%) spoke at least one time with the SBHC clinician. 

 The SBHC clinician delivered an average of 7.9 clinical sessions per participant and made phone contact with caregivers for an average of 41% of the time. Two of the four recorded encounters did not meet MI proficiency (Sessions 3 and 5) while the latter two did meet proficiency (Sessions 6 and 8). 

 In the SCG, 100% of the students met with the SBHC clinician. Sessions averaged for 28 minutes. Students rated an average satisfaction score of 4.2 (0 = not satisfied, 5 = very satisfied). 

### 3.3. Anthropometric Outcomes

 The height and weight of students in both groups increased from baseline to postintervention in these growing teenagers (Table 4). There were no between-group differences in students' height and weight. After converting height and weight to a BMI percentile for age and sex, a median decrease of 0.3% was observed in the intervention group. The SCG's BMI median percentile increased by 0.2% leading to a significant between-group difference of −0.6% (*P* = 0.04). The mean waist circumference in the intervention group remained unchanged and increased by 1.7 cm in the SCG, resulting in a significant between-group difference of −1.7 cm (*P* = 0.04). 

### 3.4. Behavioral Outcomes

 Television viewing was significantly reduced during weekdays in the intervention group by −0.4 hours/day, while the viewing time increased in the control group (0.2 hours/day) (Table 4). The median difference between the two groups was −0.7 hours/day (*P* = 0.03). 

 Physical activity as measured by the 3DPAR and RT3 accelerometer revealed no significant between-group differences. No between-group differences in total caloric intake, sweetened beverage consumption, and fruits and vegetable intake were observed (Table 4).

### 3.5. Biochemical and Blood Pressure Outcomes

 Fasting glucose increased in both groups, but the increase was smaller in the SCG (Table 5). No other significant between-group differences in changes were seen in triglycerides, HDL-cholesterol, fasting insulin, HOMA-IR, and blood pressure.

## 4. Discussion

 Obesity, especially central adiposity, is a critical contributor to the development of metabolic syndrome. High school students participating in the SBHC weight management program, ACTION, had significantly better results in BMI percentile and waist circumference compared to students receiving standard care. Waist circumference of these overweight and obese students in ACTION remained unchanged, but students in the SCG experienced an increased waist circumference over the six months. These findings are promising in light of the general challenge of weight management in adolescents and the high prevalence of type 2 diabetes (52%) and dyslipidemia (43%) in the family history of these youths, with six percent of the student participants already meeting criteria for metabolic syndrome and 43% manifesting at least one component.

 Reduced television viewing is a modifiable behavior that has been demonstrated to be associated with preventing obesity and lowering BMI [28, 29]. Students in ACTION reported reduced television viewing during weekdays compared to the standard care group. In the current study, reduction of television viewing was the only behavior that showed a significant improvement and may have contributed to the decrease in BMI percentile. 

 Hours of contact have been calculated to represent treatment intensity in obesity behavioral trials [30]. ACTION would be categorized as a very low- (<10 hours) intensity intervention. The finding of an improved BMI percentile is consistent with two other low-intensity primary care intervention trials that reported short-term outcomes and included adolescents [25, 31]. The study by Saelens and coworkers used a computer program to assess lifestyle habits and incorporated physician counseling and interaction with a behavioral specialist to learn food self-monitoring [25]. Participants were followed by telephone counselors weekly for the first eight calls and then biweekly for the last three calls, combined with three mailings designed to help adolescents acquire behavioral skills for weight control. Similar to our findings, Saelens and colleagues reported an increase in BMI among controls compared with the slight decrease of BMI among intervention participants. While their study relieves the primary care provider of some of the burden of conducting the intervention, it is not possible to determine if using behavioral specialists and telephone counselors is widely applicable to settings with limited resources such as SBHCs. Our study, however, uses the primary care provider at the SBHC to deliver all of the intervention and therefore should be generalizable to other similar clinical settings or SBHCs.

 Evidence indicates that primary care behavioral intervention trials of moderate to high intensity (>25 hours) which included adolescent participants consistently show a more beneficial effect on BMI than low-intensity interventions [32–35]. However, these interventions include components that require meetings with the physician, group meetings with a behavior specialist, regular meetings with a dietician, and/or weekly supervised exercise episodes that may not be possible in “real-world” settings. High attrition from such studies is also problematic [32, 33]. While our study demonstrated modest improvement in BMI percentile when compared to moderate- and high-intensity interventions, the potential to increase the length of the intervention over students' four year high school experience is possible and may reveal greater improvements in BMI. Additionally, because SBHCs are located on school grounds, access is more convenient than off-site primary care clinics.

 Minority status and poverty have been shown to contribute to obesity among youth because of long parental work hours [36] and a built environment that lacks access to physical activity and healthy food choices [37, 38]. Our study using SBHCs with schools serving a primarily Hispanic population has broader public health implications. Given that there are over 1,900 SBHCs in the US [39], mostly located in low-income minority communities, SBHCs may provide a focal point for efforts to intervene with overweight and obese youth. Our study provides preliminary evidence for the acceptability and short-term efficacy of such an approach for weight management. 

 One limitation of our study is the small sample size and therefore results may not be generalizable. Another limitation is the short duration of followup in this trial. While it is important to evaluate long-term efficacy of this intervention, our primary objective was to ensure the feasibility and acceptability of our SBHC approach before launching a larger and longer trial.

## 5. Conclusions

 In the first known study to evaluate the feasibility of conducting a weight management program through SBHCs, we demonstrated that a primary care clinician can be taught to use motivational interviewing and that the approach received high satisfaction scores from both the caregivers and student participants. Our findings may help to reduce obesity and prevent metabolic syndrome among adolescents who typically have limited access to preventive care [9, 40]. In minority populations where obesity, metabolic syndrome, and type 2 diabetes are disproportionately high, SBHCs warrant further research as venues to help adolescents decrease the risk of developing complications of obesity in adulthood.

## Figures and Tables

**Table 1:** General content of clinician toolkit used as a “menu of options” during clinical encounters with participants.

DVD sections:	
Adolescent motivation for change	
Strategies targeting energy balance and nutritional quality	
Physical aerobic dance and strength training	
Print materials:	
Weight loss guidelines for clinicians	
Motivational interviewing for clinicians	
Newsletter for caregivers	
Clinic displays	
Adolescent session tools (e.g., goal setting, internet resources, and activity/food journal)	

**Table 2:** Characteristics of the ACTION and standard care groups at baseline.

Characteristics	ACTION *N* = 28 *N* (%)	Standard care *N* = 23 *N* (%)	*P* value
Race/ethnicity			
Asian	4 (14%)	1 (4%)	0.10
Hispanic	21 (75%)	14 (61%)	
Native American	0 (0%)	3 (13%)	
Multiple	3 (11%)	5 (22%)	
Sex			
Female	17 (61%)	13 (57%)	0.78
Male	11 (39%)	10 (43%)	
Family history of diabetes			
No	9 (32%)	12 (52%)	0.43
Yes	17 (61%)	10 (43%)	
Don't know	2 (7%)	1 (4%)	
Family history hyperlipidemia			
No	9 (32%)	9 (39%)	0.88
Yes	12 (43%)	10 (43%)	
Don't know	7 (25%)	4 (17%)	
Caregiver years of education			
0–6	3 (11%)	2 (9%)	0.99
7–11	8 (29%)	8 (35%)	
12 (high school graduate)	6 (21%)	5 (22%)	
13–15	7 (25%)	6 (26%)	
16 or more	4 (14%)	2 (9%)	
Caregiver preferred language			
Spanish	10 (36%)	10 (43%)	0.11
English	13 (46%)	12 (52%)	
English and Spanish	5 (18%)	0 (0%)	
English and other	0 (0%)	1 (4%)	
Metabolic syndrome components^a^			
Large waist circumference (Men: ≥102 cm; Women: ≥88 cm)	2 (7%)	3 (13%)	0.65
High blood pressure (Systolic ≥130 and/or diastolic ≥85 mm Hg)	0 (0%)	0 (0%)	—
Low HDL cholesterol (Men: <40 mg/dL; Women: <50 mg/dL)	5 (18%)	6 (26%)	0.51
Elevated triglycerides (≥150 mg/dL)	8 (29%)	6 (26%)	1.00
Elevated fasting blood glucose (≥100 mg/dL)	0 (0%)	0 (0%)	—
Stages of change			
Precontemplation	0 (0%)	3 (13%)	0.22
Contemplation	4 (14%)	1 (4%)	
Action	16 (57%)	13 (57%)	
Maintenance	8 (29%)	6 (26%)	

^a^Defined by the National Heart, Lung, and Blood Institute.

**Table 3:** Anthropometric measurements of ACTION and standard care groups at baseline.

Characteristics	ACTION *N* = 28	Standard care *N* = 23	
	Mean (SD)	Mean (SD)	*P* value
Height (cm)	164.4 (8.1)	163.1 (10.9)	0.64
Weight (kg)	78.5 (12.5)	78.1 (18.1)	0.92
BMI percentile	94.5 (4.1)	94.4 (4.6)	0.94
Waist circumference (cm)	89.9 (8.5)	89.9 (9.1)	1.00

**Table 4:** Comparison of anthropometric and behavioral changes between ACTION and standard care groups.

Measures	ACTION group *N* = 28	Standard care group *N* = 23	Between-group difference	
	Mean (95% CI)	Mean (95% CI)	Mean (95% CI)	*P* value
Height (cm)				
Pre	164.4 (161.2, 167.5)	163.1 (158.4, 167.8)		
Post	165.0 (161.7, 168.2)	164.1 (159.1, 169.0)		
Difference	0.6 (0.0, 1.2)	1.0 (0.3, 1,6)	−0.4 (−1.2, 0.5)	0.31
Weight (kg)				
Pre	78.5 (73.7, 83.4)	78.1 (70.3, 85.9)		
Post	80.2 (74.9, 85.6)	80.6 (72.5, 88.8)		
Difference	1.7 (0.2, 3.2)	2.5 (0.8, 4.3)	−0.8 (−3.1, 1.4)	0.12
BMI percentile^d^				
Pre	97.0 (92.8, 97.4)	96.2 (91.6, 97.8)		
Post	96.3 (92.1, 97.4)	96.1 (91.9, 98.5)		
Difference	−0.3 (−0.6, 0.3)	0.2 (−0.1, 0.8)	−0.6 (−1.2, 0.1 )	0.04
Waist circumference (cm)				
Pre	89.9 (86.6, 93.2)	89.9 (84.9, 94.8)		
Post	89.9 (86.3, 93.4)	91.5 (86.5, 96.5)		
Difference	0.0 (−1.4, 1.4)	1.7 (0.4, 2.9)	−1.7 (−3.6, 0.2)	0.04
YAQ^a^ calories/day				
Pre	1916 (1807, 2225)	2270 (1852, 2687)		
Post	2086 (1700, 2473)	2017 (1642, 2392)		
Difference	170 (−300, 641)	−252 (−729, 224)	422 (−239, 1084)	0.21
YAQ^a^ sweetened drinks (glasses/day)^d^				
Pre	0.76 (0.34, 1.08)	0.58 (0.30, 0.79)		
Post	0.19 (0.15, .44)	0.43 (0.16, 0.59)		
Difference	−0.12 (−0.47, −0.08)	−0.16 (−0.57, 0.22)	−0.08 (−0.57, 0.41)	0.23
YAQ^a^ fruits and vegetables (servings/day)^d^				
Pre	2.81 (2.06, 4.36)	1.91 (1.70, 3.52)		
Post	2.48 (2.04, 3.44)	1.71 (1.46, 2.12)		
Difference	−0.22 (−0.72, 0.41)	−1.16 (−0.56, −0.02)	0.42 (−0.32, 1.26 )	0.47
MVPA^b^ by 3DPAR^c^ (30 minute blocks/day)^d^	*N* = 27	*N* = 20		
Pre	1.4 (0.7, 3.7)	2.0 (1.0, 3.3)		
Post	1.7 (0.7, 3.3)	1.0 (0.0, 4.0)		
Difference	0.0 (−2.0, 0.7)	−0.9 (−1.3, 0.4)	0.6 (−1.6, 2.0)	0.63
MVPA^b^ by accelerometer (mins/day)	*N* = 14	*N* = 8		
Pre	49.4 (37.7, 61.2)	64.7 (41.8, 87.6)		
Post	65.9 (47.6, 84.2)	57.6 (39.9, 75.4)		
Difference	16.5 (−2.8, 35.8)	−7.1 (−22.9, 8.7)	23.6 (−3.4, 50.5 )	0.08
Television weekday viewing (hours/day)^d^				
Pre	1.8 (1.0, 2.4)	1.6 (1.4, 2.2)		
Post	1.0 (0.6, 1.4)	1.8 (1.1, 2.4)		
Difference	−0.4 (−1.0, 0.2)	0.2 (−0.3, 0.6)	−0.7 (−1.6, 0.0)	0.03
Television weekend viewing (hours/day)^d^				
Pre	1.9 (1.5, 3.0)	2.2 (1.5, 5.5)		
Post	1.6 (0.8, 3.0)	3.0 (2.2, 3.0)		
Difference	−0.1 (−0.8, 0.0)	0.5 (−2.0, 1.5)	−0.6 (−1.9, 1.3)	0.17

^a^YAQ: Youth Adolescent Questionnaire food frequency survey.

^b^MVPA: moderate or vigorous physical activity.

^c^3DPAR: 3-Day Physical Activity Recall.

^d^Results are reported as median (95% CI). Wilcoxon rank sum test was used for *P* value and bootstrap procedure was used for confidence intervals.

**Table 5:** Comparison of biochemical measures between ACTION and standard care groups.

Measures	ACTION group *N* = 28	Standard care group *N* = 23	Between-group difference	
	Mean (95% CI)	Mean (95% CI)	Mean (95% CI)	*P* value
HDL-C (mmol/L)				
Pre	1.07 (0.98, 1.16)	1.06 (0.97, 1.16)		
Post	1.07 (0.98, 1.17)	1.03 (0.9, 1.13)		
Difference	0.0 (−0.09, 0.09)	−0.04 (−0.09, 0.02)	0.04 (−0.07, 0.14)	0.50
Triglycerides (mmol/L)				
Pre	1.4 (1.2, 1.7)	1.3 (1.0, 1.5)		
Post	1.5 (1.1, 1.9)	1.4 (1.1, 1.6)		
Difference	0.1 (−0.3, 0.4)	0.1 (−0.1, 0.3)	0.0 (−0.4, 0.4)	0.95
Fasting glucose (mmol/L)				
Pre	4.5 (4.4, 4.7)	4.7 (4.6, 4.8)		
Post	4.8 (4.7, 4.9)	4.8 (4.6, 4.9)		
Difference	0.3 (0.1, 0.4)	0.1 (−0.1, 0.2)	0.2 (0.03, 0.4)	0.04
Fasting insulin (pmol/L)^d^				
Pre	93.4 (69.4, 134.0)	96.5 (67.4, 112.5)		
Post	85.1 (67.4, 112.5)	83.3 (67.4, 120.1)		
Difference	−10.1 (−23.8, 34.4)	1.4 (−19.4, 25.0)	−5.7 (−39.6, 40.3)	0.59
HOMA-IR^a,d^				
Pre	2.6 (1.9, 3.9)	3.1 (2.1, 4.1)		
Post	2.5 (2.0, 3.6)	2.5 (2.0, 3.6)		
Difference	0.0 (−0.6, 1.1)	0.7 (−0.7, 2.1)	−0.1 (−0.6, 0.9)	1.00

^a^HOMA-IR: homeostatic model assessment insulin resistance index.

^d^Results are reported as median (95% CI). Wilcoxon rank sum test was used for *P* value and bootstrap procedure was used for confidence intervals.

## Abbreviations

SBHC: School-based health center
ACTION: Adolescents Committed to Improvement of Nutrition and Physical Activity
SCG: Standard care group
MI: Motivational interviewing
YAQ: Youth Adolescent Questionnaire
MVPA: Moderate or vigorous physical activity
METs: Metabolic equivalents.
